# The Science-spirituality Nexus: Religion and the COVID-19 vaccination campaigns in Tanzania

**DOI:** 10.4102/jphia.v16i3.706

**Published:** 2025-04-18

**Authors:** Richard F. Sambaiga, Chima E. Onuekwe, Tumaini Haonga, William Mwengee

**Affiliations:** 1 Department of Sociology and Anthropology, College of Social Sciences, University of Dar es Salaam, Dar es Salaam, United Republic of Tanzania; 2 Department of Immunization, Emergency Preparedness and Response, World Health Organization, Dar es Salaam, United Republic of Tanzania; 3 Centre for Health and Allied Legal and Demographical Development, Research and Training, Nnamdi Azikiwe University, Awka, Nigeria; 4 Health Promotion Unit, Ministry of Health, Dodoma, United Republic of Tanzania

**Keywords:** religion, COVID-19 vaccination, health promotion, hesitancy, uptake

## Abstract

**Background:**

The influence of religion on health seeking behaviour is well document in the public health literature. However, the extent to which religious discourses and practices contributed to scepticism towards COVID-19 vaccines, vaccine uptake, and indecisiveness in intention to be vaccinated in Tanzania has not yet been established.

**Aim:**

To explore the nexus between religion and public health in the measures taken against the COVID-19 pandemic in Tanzania by empirically examining how religious actors in opposed the first phased of COVID-19 vaccination campaigns before becoming key supporters of the same campaigns in the second phase.

**Setting:**

The study was conducted in eight regions representing key administrative zones of Mainland Tanzania.

**Methods:**

The article draws on empirical evidence from exploratory mixed-method study combining focus group discussions (FGDs), key informant and semi-structured interviews.

**Results:**

We found that religious narratives and practices in relation to the pandemic were quite dynamic but influential in shaping individuals’ decisions including on whether or not to take the COVID-19 vaccine. Religious anti-COVID-19 vaccine narratives accounted for the slow COVID-19 vaccine uptake but when religious leaders were later mobilised to support the COVID-19 vaccination campaign, the vaccine uptake in Tanzania improved considerably.

**Conclusion:**

The study concludes that religious actors play a significant role in influencing public health behaviours, particularly in vaccine uptake.

**Contribution:**

Future public health measures designed to increase vaccine uptake should not overlook the salient role of religious actors in the promotion desired health practices and outcomes.

## Introduction

Delay in acceptance or refusal of vaccines despite availability of vaccine services commonly referred to as vaccine hesitancy is not a new public health phenomenon in global health. It is among other factors accounting for the current low COVID-19 vaccine coverage in Africa^1,2^ just like in many low- and middle-income countries (LMICs). Other structural constraints include geopolitical influence and economic interests of vaccine providers compromising vaccine acceptance by attracting sceptics, misinformation from social media and religious groups.^1^ There is a growing global health literature calling for cultural sensitive COVID-19 vaccination programmes in sub-Saharan Africa in order to address drivers for low rates of COVID-19 vaccination and unwillingness to accept the vaccine.^2^

Prominent scholars in religious studies have represented Africa as deeply religious and faith-based beliefs generally affect the practice of everyday life in the continent.^3^ Available literature observes that religion has been actively steering vaccine resistance in majority of African communities.^4,5^ This is precisely because religious beliefs influence health-seeking behaviours of believers. Banerjee et al.^6^ underline the salience of religious actors in public health promotion highlighting that faith and traditional leaders hold significant influence within their communities to influence the acceptance of vaccination.^7,8^ Religious writings and scripts contain references and quotations on health and well-being, which individuals and communities interpret differently perhaps negatively towards vaccination.^9^

Ruijs et al.^10^ highlights three typologies of how religious leaders engage with contentious vaccination. Firstly, those who fully accept vaccination and do not address the topic. Secondly, those who have religious objections to vaccination but focus on deliberate choice. Thirdly, those who have religious objections to vaccinations and preach against vaccination.^10^ Religious rituals and traditional practices such as prayers, faith healing and offerings may be seen as alternative or complementary avenues for seeking protection from disease.^11,12,13,14^ However, religious narratives and practices negatively affect the outcomes of vaccination campaigns in many African countries.^15,16^ Thus, addressing vaccine hesitancy in the African contexts requires engaging religious and traditional leaders to generally build or restore public confidence on the safety and efficacy of vaccines.^17,18^

In Tanzania, religious leaders and religious institutions played a crucial role in shaping the outcome of the implementation of the COVID-19 standard guidelines issued by the government of Tanzania through the Ministry of Health.^19^ However, the extent to which religious discourses and practices contributed to scepticism towards COVID-19 vaccines, vaccine uptake and indecisiveness in intention to be vaccinated in Tanzania has not yet been established. This is despite the fact that COVID-19 vaccination campaigns were rife with controversies involving competing religious and public health discourses. Thus, in this article, we focus on the dominant religious narratives about COVID-19 to examine how religious actors in Tanzania opposed the first phase of COVID-19 vaccination campaigns before they became key supporters of the same campaigns in the second phase. We argue that dynamics in religious discourses on the pandemic and its vaccination are reflective of the exponential increase of COVID-19 vaccination coverage to 52.5% by May 2023 from a poor coverage of 2.8% by January 2022.^20^

Conceptually, we draw inspiration on the Health Belief Model (HBM), one of the public health perspectives informing the prediction of health behaviours in a given population including preventive health behaviours such as vaccination uptake and adherence to recommended medical regimens.^21^ The model focusses on both individuals’ conceptions of health and health behaviour, which entail threat perception and behavioural evaluation. While threat perception is considered to be a product of perceived susceptibility to health problems and anticipated severity of the consequences, behavioural evaluation entails beliefs about the benefits or efficacy of a recommended health action such as taking a COVID-19 vaccination, and the costs of, or barriers to, enacting the behaviour.^21^ The model further acknowledges the role of such triggers or cues as individual perceptions of symptoms, social influence and health promotion education campaigns. The multiple elements addressed by the HBM are pertinent for any attempt to better understand the role of religious beliefs and practices in shaping the dynamics in the uptake of the COVID-19 vaccine in Tanzania.

## Research methods and design

This article draws on a cross-sectional large-scale mixed-method study conducted in eight regions of Mainland Tanzania from June 2023 to August 2023. Representing key administrative zones of Tanzania, the study regions included Arusha, Mbeya, Morogoro, Mtwara, Njombe, Shinyanga, Singida and Tabora. Three councils were selected from each of the study regions considering the rural–urban divide. Given the exploratory nature of the study, purposive sampling techniques were used to select participants and respondents for qualitative and quantitative inquiries, respectively. Key informant interviews (KIIs), focus group discussions (FGDs) and semi-structured interviews were conducted. Six FGDs with 8–12 participants, and KIIs were conducted per region making a total of 42 FGDs and 42 KIIs. Key informant interviews intended to capture view and perspectives on the role of religion in promoting or/and constraining the update of COVID-19 vaccine from such actors as political and religious leaders, leaders of community groups and/or associations, opinion leaders, civil servants such as teachers, medical practitioners and traditional birth attendants. Likewise, FGDs were conducted with participants from the study groups to grasp shared understanding and collective opinion on the key study aspects. Both FGDs and KIIs were recorded subject to securing the consent of participants. Qualitative data were complemented with semi-structured interviews with a total of 3099 respondents to establish key patterns and associations between demographic, social and cultural factors on the knowledge, attitude and practices towards the COVID-19 vaccine. While both content and thematic analysis were conducted for qualitative data, descriptive and inferential analysis mainly Chi-square test was performed for quantitative data to determine the association between religion and the COVID-19 vaccine uptake.

### Ethical considerations

Ethical approval to conduct this study was obtained from The University of Dodoma Institutional Research Review Committee (IRREC) (No. MA.84/261/76/214).

## Results

We present both qualitative and quantitative findings on three key dimensions demonstrating the role of religion shaping the public health interventions and outcomes during the COVID-19 pandemic in Tanzania. Specifically, we demonstrate how religion shapes the knowledge about COVID-19, the prevention practices and ultimately the pattern and trend of uptake of COVID-19 vaccine as delineated in the subsequent sections. The demographic characteristics of the participants are presented in Table 1 prior to the findings.

**TABLE 1:** Demographic characteristics of respondents (*N* = 3099).

Variable	Category	*n*	%
Region	Arusha	358	11.55
	Mbeya	431	13.91
	Morogoro	365	11.78
	Mtwara	420	13.55
	Njombe	391	12.62
	Shinyanga	369	11.91
	Singida	386	12.46
	Tabora	378	12.20
	No response	1	0.03
Age (years)	18–29	689	22.23
	30–39	871	28.11
	40–49	645	20.81
	50–59	438	14.13
	60 and above	456	14.71
Gender	Female	1757	56.70
	Male	1341	43.27
	Prefer not to say	1	0.03
Religion	Christian	1837	59.28
	Muslim	1198	38.66
	Others	64	2.07
Educational level	No formal education	296	9.55
	Primary education	1917	61.86
	Secondary education	699	22.56
	University/tertiary	114	3.68
	Vocational education	73	2.36
Employment status	Employed – Government	67	2.16
	Employed – Private	171	5.52
	Retired	105	3.39
	Self-employed	2131	68.76
	Unemployed	625	20.17
Marital status	Divorced/Separated	169	5.45
	Married	2067	66.70
	Single	624	20.14
	Widowed	234	7.55
	Other	5	0.16
Income level per month (in TZS)	Above 100 000–250 000	1000	32.27
	Above 1m	21	0.68
	Above 250 000–500 000	276	8.91
	Above 500 000–1m	74	2.39
	Less than 100 000	1728	55.76
Where do you live?	Rural	1343	43.34
	Semi-urban	779	25.14
	Urban	977	31.53

TZS, Tanzanian shilling.

Table 1 depicts the diversity of the research subjects involved in the study even though the main focus of the present analysis in the role of religion in shaping various aspects of health-seeking practises against COVID-19. The Chi-square tests were computed to measure the association between knowledge about COVID-19 and religion. The findings showed that Christians (76.7%) were relatively more knowledgeable on handshaking as factor that can lead to transmission, compared to Muslims (69.45%). Respondents from other religions and atheist had the lowest level of knowledge mainly because of their late exposure to public health measures compared to their counterparts (Table 2).

**TABLE 2:** Religious association with different aspects of COVID-19.

Variables	Christians (%)	Muslims (%)	Other religions/ atheist (%)	*χ* ^2^	*P*
**Knowledge on COVID-19 transmission**	-	-	-	-	-
Handshaking infected person	76.7	69.45	56.25	29.517	0.000
Contact with infected person	43.33	42.4	20.31	13.417	0.001
Touching surfaces infected	32.23	26.21	12.5	21.696	0.000
Breath, coughs or sneezes	73.76	71.87	48.44	20.295	0.000
I don’t know	8.22	11.27	31.25	41.128	0.000
**Have received COVID-19 vaccination**	-	-	-	24.999	0.000
Yes	59.23	38.73	2.05	-	-
No	66.67	28.57	4.76	-	-
**Preventive measures**	-	-	-	-	-
Regular handwashing	78.66	76.46	64.06	16.165	0.000
Social distancing	56.78	51.75	17.19	30.884	0.000
Vaccination	19.98	23.96	7.81	10.229	0.006
Face masking	69.84	66.94	39.06	3.014	0.222
I don’t know	4.57	5.59	12.5	16.165	0.000
**Belief in effectiveness of COVID vaccine**	-	-	-	36.371	0.000
Strongly agree	14.21	17.7	6.25	-	-
Agree	30.16	34.89	40.62	-	-
Neutral	40.88	36.31	46.88	-	-
Disagree	8.6	7.76	6.25	-	-
Strongly disagree	6.15	3.34	0	-	-

Table 3 presents the results of odds ratio (OR) between COVID-19 vaccine uptake and religion. The results show that Muslims were found to have at least 1.45 times (crude odds ratio [COR] = 1.45, 95% confidence interval [CI]: 1.25, 1.69, *p* < 0.00), even after adjusting for covariates (adjusted odds ratio [AOR] = 1.44, 95% CI: 1.24, 1.67, *p* < 0.00). The COR showed that believers in other religions and atheist had higher chance of being vaccinated compared to Christians, although these findings were not statistically significant. This suggests that there is not enough evidence to conclude the likelihood of more believers in traditional religions and atheists being vaccinated than Christians. Despite, this, the findings imply that, religion played an important role in shaping COVID-19 vaccine uptake. This could be because of different religious beliefs linked to vaccine and the COVID-19 pandemic as a whole.

**TABLE 3:** Religious influence on COVID-19 vaccine uptake.

Independent variable	COR	95% CI	*P*	AOR	95% CI	*P*
Christian	0.51	0.46–0.56	0	0.76	0.59–0.97	0.03
Muslim	1.45	1.25–1.69	0	1.44	1.24–1.67	0.00
Other religions and atheist	0.89	0.52–1.52	0.66	0.75	0.43–1.31	0.31

COR, crude odds ratio; AOR, adjusted odds ratio; CI, confidence interval.

The findings from the study are further corroborated by qualitative data gathered from in-depth interviews and FGDs with Christian and Islamic religious leaders who not only confirmed an understanding of the ways of transmission but also the symptoms of the disease in line with the public health discourse. The following extracts serve to illuminate how religious leaders responded to various COVID-19 campaigns in Tanzania:

‘We were told that COVID-19 is being spread through the flu, body fluids, and touching the person who has already been infected.’ (KII5, Christian religious leader, Shinyanga)

‘I heard many things about COVID-19, and I was told it is a disease that is in our country, and it spreads through the air, through sneezing and shaking hands.’ (KII7, Christian religious leader, Singida)

‘I vaccinated twice, and I have a certificate … I know it can be infected through flu, coughing, and even wind … we also installed a basket for washing hands before entering the mosque.’ (KII13, Islamic religious leader, Tabora)

‘COVID-19 was a serious disease, and its symptoms as described by health workers include severe colds, chest tightness, and other symptoms …’ (KII18, Islamic religious leader, Arusha)

‘Even the church prohibited overcrowding … a distancing seating arrangement and encouraged the wearing of masks …’ (KII26, Village Chairperson, AKerli-Meru)

The above-stated comments from religious leaders reflect the degree to which public health messages on COVID-19 were received, internalised, disseminated and applied by religious leaders, and by extension, their congregations. It is important therefore to argue that religion played a supportive role in promoting public health awareness on the pandemic in Tanzania. Both Christian and Islamic religious leaders used their houses of worship to not only disseminate public health messages about the pandemic but also demonstrated how to comply with preventive measures. Thus, besides appealing to prayers, the leaders demonstrated a better understanding of the public health guidelines and measure against the pandemic.

Having adequate knowledge on how to prevent from a disease is often expected to lead into actual preventive actions on the part of individuals and communities. It was found that the majority of the respondents from both the different religious background reported to have applied measures such as wash their hands regularly and wearing face masks in crowded places in order to prevent themselves from COVID-19. This was followed by those who reported social distancing. However, less than a quarter of the respondents got vaccinated as illustrated in Table 2.

### Religion and the uptake of COVID-19 vaccine

Tanzania registered an interesting trend in terms of the coverage of COVID-19 vaccination. Despite the fact that it remains relatively low in coverage, the trend of COVID-19 vaccination recorded a steady increase in coverage by the year 2023 after almost 2 years of stagnation.^20^ Although multiple factors explain the aforesaid trend in vaccination, we argue that religion played a fundamental role in promoting the COVID-19 vaccination campaign in Tanzania.

In line with the HBM, individuals and communities are more likely to accept a vaccine or any other medication if they believe that it is effective in addressing the health problem, and the vice versa is true. We found mixed picture but suggestive of the pattern of vaccination portrayed in the previous section. While some of the respondents were not sure of the efficacy of COVID-19 vaccine in terms of preventing severe illness or death as demonstrated, some confirmed to have confidence in the vaccine, and yet others completely rejected the idea that the vaccine can be of any possibility of efficacy in the vaccine as presented in Table 4.

**TABLE 4:** Belief on the effectiveness of COVID-19 vaccines by religion.

Religion	Effectiveness of COVID-19 vaccines (%)					*χ* ^2^	*P*
	Strongly agree	Agree	Neutral	Disagree	Strongly disagree		
Christian	14.21	30.16	40.88	8.6	6.15	36.371	0.000
Muslim	17.7	34.89	36.31	7.76	3.34	-	-
Other	6.25	40.62	46.88	6.25	0.00	-	-

The findings presented in Table 4 indicate that a large proportion of the surveyed respondents were not sure of the efficacy of the vaccine. However, the proportion of respondents who did not believe in the effectiveness of the vaccine was relatively low compared to the other two categories. The pattern of response was consistent across the religious affiliation. In line with the HBM, the findings suggest the presence of competing beliefs on the effectiveness of the vaccines, which explains the trends and pattern of the COVID-19 vaccine hesitancy and uptake in Tanzania.

### Religious narratives on COVID-19 and its vaccine

The discursive practice followed by selected religious leaders in Tanzania in response to the pandemic and associated public health measures sheds light on the dynamics of vaccine hesitancy and uptake in the country. Indeed, the slow COVID-19 vaccine uptake as well as subsequent improvement should be partly explained by the pattern of religious discourses on COVID-19 in the country.

Initially, when COVID-19 vaccines were not yet available in the country, the focus of religious discourses was appealing to prayers alongside compliance with instructions from public health experts. The only area of controversy was whether the country should adopt lockdown measures recommended by World Health Organization (WHO) as a means to reduce the pace at which the pandemic was spreading. When the government of Tanzania boldly resisted total lockdown (temporarily closed schools only), religious leaders supported government decisions and organised a series of prayer sessions asking for protection upon the country and God’s intervention. Of course, there were few exceptions of religious leaders who objected government decisions and went ahead to lockdown their own churches:

‘Listen to me, I now know who is paying attention to the word of God, you are full of fear to extent of coming to church covering your mouth how will you speak to God? Is God smelling?’ (TV1, Christian religious leader, Televised Sunday services)

‘Now there are even Pastors claiming that they can deal with COVID-19 just in the name of Jesus … listen to me I cannot make jokes with God I should play my part.’ (TV2, Christian religious leader, Dar es Salaam)

‘Imagine just because of COVID-19 they are blaming the President for allowing churches to resume their worshiping sessions.’ (TV3, Christian religious leader, Televised Sunday service)

The arrival of COVID-19 vaccines in Tanzania alongside campaigns for and against it globally and within the country shaped religious narratives on the vaccine. The shift in government’s approach towards the pandemic was evident following the passing on of the fifth President of Tanzania, John Magufuli, in March 2021. Unlike the fifth government, the sixth government launched explicit countrywide COVID-19 vaccination campaigns. Few religious leaders continued to preach against the vaccine, some supported the campaigns, while others silently ignored them or were simply confused or in dilemma. The following empirical findings from FGDs and in-depth interviews with members of religious groups and religious leaders illuminate the aforesaid discursive dynamics.

During a FGD conducted in Oldonyo Sambu in Arusha region, participants underlined that religious leaders’ preaching against COVID-19 vaccine emphasised that the aetiology of the disease has a lot to do with evil spirits. As such, prayers should be considered the right measure, which in turn implied that there is no need to be vaccinated. ‘People should not be vaccinated because COVID-19 is just a demon to be that can be casted with prayers’ (FGD, Oldonyo Sambu, Arusha) was a key message from some of the religious leaders. Participants confirmed that because the pastor is not vaccinated, so did his church members.

Views from participants and informants consulted in Mbeya region reflected the shifts in the COVID-19 discourse in Tanzania. A religious leader from Kyela asserted:

‘Things that were said in the past are what made me not get the COVID-19 vaccine … we heard things like the side effects of vaccine that it can damage your body some years later.’ (KII30, Religious leader, Kyela)

Reflecting on the nexus between political and religious discourse on the pandemic, the religious leader asked ‘Why were these vaccine campaigns introduced after the passing on of President Magufuli?’ (KII30, Religious leader, Kyela). The question was alluding to changes in the government approach against the pandemic.

Similar sentiments were captured in Shinyanga and Tabora region whereby religious leaders were not yet convinced by the public health recommendations regarding the COVID-19 vaccine. While some considered religious explanatory model of disease and death to outweigh the public health narrative, others felt that they were more exposed to the anti-COVID-19 vaccine messages. A few examples serve to illustrate this point. A Muslim religious leader from Shinyanga stated ‘I haven’t had enough information to convince me that the vaccination is safe’ (KII3, Islamic religious leader, Shinyanga District Council).

In sharp contrast to the aforesaid, a pastor from Tabora objected vaccination not because he doubted its efficacy but because of his inner spiritual hesitations. To that end he noticed ‘It’s not that the vaccine is harmful but it’s just that I didn’t have the peace of being vaccinated’ (KII10, pastor of Free Pentecostal Church of Tanzania [FPTC] Church, Uyui, Tabora). It is on the basis of his spiritual conviction, the pastor concluded that the COVID-19 vaccination does not protect people from death.

Clearly, the predominately anti-COVID-19 vaccine discourses as highlighted greatly induced hesitancy towards the vaccine. However, in order to promote uptake of the COVID-19 vaccine, it was imperative to engage religious actors and seek their support on the COVID-19 vaccination campaign. In what follows, we emphasise empirical evidence to illuminate how religion supported public health campaign for COVID-19 vaccine in Tanzania.

Across the study regions in Tanzania, religious leaders from different religious background attested various ways in which they got engaged in the COVID-19 vaccination campaigns. A pastor in Singida reported to have accommodated COVID-19 prevention measured within the church. During the vaccination campaigns, the COVID-19 vaccination campaign team was invited to his church on a Sunday mass where he claimed ‘I urged the church members to be vaccinated and most of them were vaccin1ated on that Sunday’ (KII12, Christian religious leader, Ikungi-Ihanja, Singida).

A religious leader interviewed in Mtwara region further asserted; ‘In our preaching, for example, at the Friday gathering, we were giving the education that people should get vaccinated because this disease is very dangerous …’ (KII40, Islamic religious leader, Mtwara DC). Similar expressions were captured in an interview with an informant in Shinyanga region who hinted ‘when the COVID-19 vaccination campaign was introduced, religious leaders were given flyers to publicize in their congregation in order to deliver the message to the community’ (KII16, Economic group leader, Shinyanga District Council).

## Discussion

The purpose of this study was to understand the nexus between religion and public health in the fight against the COVID-19 pandemic. The empirical evidence from Tanzania confirms that religious discourses and practices can promote and/or constrain success of public health measures. In line with the HBM, the study found that religious narratives and responses in relation to the pandemic were quite dynamic but critical in shaping individuals’ decisions including on whether or not to take the COVID-19 vaccine. This is also reflected in a growing literature on the role of religion in supporting^17,18,22^ and challenging^4,23^ public health interventions against the COVID-19 pandemic.

In the Tanzanian context, the study suggests that religion was used as a powerful vehicle through which public health messages on COVID-19 were received, disseminated and applied by religious leaders and their congregations. This was evidenced by a relatively better understanding of the ways in which COVID-19 can be prevented among respondents regardless of the religious background. The knowledge on COVID-19 prevention was actually translated into preventive actions such washing their hands regularly, wearing face masks and social distancing in crowded places including in churches and mosques. This corroborates the findings by Wagana^18^ confirming that religious leaders actively participated in the implementation of the COVID-19 standard guidelines issued by the government of Tanzania through the Ministry of Health.^19^ Our findings are also consistent with credible observation by Thinane^24^ that almost all major religions in the world support public health measures as long as they comply with religious precepts for the preservation, protection or well-being of livelihoods.

Nevertheless, COVID-19 vaccination was among the highly contentious public health measure for religious leaders and followers to accommodate because of the beliefs attached to the efficacy of the vaccine. Religious anti-COVID-19 vaccine narratives accounted for the slow COVID-19 vaccine uptake so much so that it was inevitable to mobilise the support of religious leaders in the COVID-19 vaccination campaign that managed to improve uptake of the vaccine in Tanzania. Other studies have also documented strong relationship between lower or higher uptake of vaccines and religious affiliation.^16,25,26,27,28,29^

The exploratory nature of the study design necessitates the use of non-probability sampling, which limits the generalisability of the findings even within the study areas not least beyond the study region. Nevertheless, the findings shed light on the ambivalent role of religion in shaping vaccine uptake and hesitancy.

## Conclusion

Religious discourses on the COVID-19 pandemic and its vaccine have greatly affected the trend of COVID-19 vaccine in Tanzania. The findings of this study suggest that public health measures designed to increase vaccine uptake should not overlook the salient role of religious actors in promoting or/and constraining desired health practices and outcomes.

## References

[1] Mutombo CS, Bakari SA, Ntabaza VN, et al. Perceptions and use of traditional African medicine in Lubumbashi, Haut-Katanga province (DR Congo): A cross-sectional study. PLoS One. 2022;17(10):e0276325. 10.1371/journal.pone.0276325 36256659 PMC9578634

[2] Ajeigbe O, Arage G, Besong M, et al. Culturally relevant COVID-19 vaccine acceptance strategies in sub-Saharan Africa. Lancet Glob Health. 2022;10(8):e1090–e1091. 10.1016/S2214-109X(22)00251-0 35691328 PMC9183213

[3] Mbiti JS. Introduction to African religion. 2nd ed. Portsmouth: Heinemann Educational Books; 1991.

[4] Costa JC, Weber AM, Darmstadt GL, Abdalla S, Victora CG. Religious affiliation and immunization coverage in 15 countries in sub-Saharan Africa. Vaccine. 2020;38(5):1160–1169. 10.1016/j.vaccine.2019.11.024 31791811 PMC6995994

[5] Oduwole EO, Mahomed H, Laurenzi CA, Larson HJ, Wiysonge CS. Point-of-care vaccinators’ perceptions of vaccine hesitancy drivers: A qualitative study from the cape metropolitan district, South Africa. Vaccine. 2021;39(39):5506–5512. 10.1016/j.vaccine.2021.08.054 34446319

[6] Banerjee P, Seth R, Dhaliwal BK, et al. Vaccine acceptance in rural India: Engaging faith leaders as vaccine ambassadors. Front Public Health. 2022;10:979424. 10.3389/fpubh.2022.979424 36203681 PMC9531688

[7] Essa-Hadad J, Abed Elhadi Shahbari N, Roth D, Gesser-Edelsburg A. The impact of Muslim and Christian religious leaders responding to COVID-19 in Israel. Front Public Health. 2022;10:1061072. 10.3389/fpubh.2022.1061072 36582370 PMC9792761

[8] Grabenstein JD. What the world’s religions teach, applied to vaccines and immune globulins. Vaccine. 2013;31(16):2011–2023. 10.1016/j.vaccine.2013.02.026 23499565

[9] Williams JT, Fisher MP, Bayliss EA, Morris MA, O’Leary ST. Clergy attitudes toward vaccines and vaccine advocacy: A qualitative study. Hum Vaccin Immunother. 2020;16(11):2800–2808. 10.1080/21645515.2020.1736451 32209009 PMC7734036

[10] Ruijs WL, Hautvast JL, Van IJzendoorn G, Van Ansem WJ, Van der Velden K, Hulscher ME. How orthodox protestant parents decide on the vaccination of their children: A qualitative study. BMC Public Health. 2012;12:408. 10.1186/1471-2458-12-408 22672710 PMC3434025

[11] Levin J, Idler EL, VanderWeele TJ. Faith-based organizations and SARS-CoV-2 vaccination: Challenges and recommendations. Public Health Rep. 2022;137(1):11–16. 10.1177/00333549211054079 34694939 PMC8721765

[12] Jalloh MF, Bennett SD, Alam D, et al. Rapid behavioral assessment of barriers and opportunities to improve vaccination coverage among displaced Rohingyas in Bangladesh, January 2018. Vaccine. 2019;37(6):833–838. 10.1016/j.vaccine.2018.12.042 30642728 PMC10472973

[13] Owoyemi A, Okolie EA, Omitiran K, et al. Importance of community-level interventions during the COVID-19 pandemic: Lessons from Sub-Saharan Africa. Am J Trop Med Hyg. 2021;105(4):879. 10.4269/ajtmh.20-1533 34370697 PMC8592170

[14] Ha W, Salama P, Gwavuya S, Kanjala C. Is religion the forgotten variable in maternal and child health? Evidence from Zimbabwe. Soc Sci Med. 2014;118:80–88. 10.1016/j.socscimed.2014.07.066 25108694

[15] Olivier J. Interventions with local faith communities on immunization in development contexts. Rev Faith Int Affairs. 2016;14(3):36–50. 10.1080/15570274.2016.1215843

[16] Eriksson K, Vartanova I. Vaccine confidence is higher in more religious countries. Hum Vaccin Immunother. 2022;18(1):1–3. 10.1080/21645515.2021.1883389 PMC8920254 33705261

[17] Njoga EO, Awoyomi OJ, Onwumere-Idolor OS, Awoyomi PO, Ugochukwu ICI, Ozioko SN. Persisting vaccine hesitancy in Africa: The whys, global public health consequences and ways-out – COVID-19 vaccination acceptance rates as case-in-point. Vaccines. 2022;10:1934. 10.3390/vaccines1011193 36423029 PMC9697713

[18] Wagana P. The role of religion in response to COVID-19 pandemic challenges in Tanzania. In: Sibanda F, Muyambo T, Chitando E, editors. Religion and the COVID-19 Pandemic in Southern Africa. 1st ed. London: Routledge, 2022; p. 115–125.

[19] WHO. From below 10 to 51 percent – Tanzania increases COVID-19 vaccination coverage [homepage on the Internet]. 2023 [cited 2024 Feb 10]. Available from: https://www.afro.who.int/countries/united-republic-of-tanzania/news/below-10-51-percent-tanzania-increases-covid-19-vaccination-coverage

[20] Conner M, Norman P. Predicting health behavior: Research and practice with social cognition models. 2nd ed. Maidenhead: Open University Press; 2005.

[21] Ebenezer D. Religion, trust in government and COVID-19 vaccine acceptance in Africa. J Soc Sci. 2023;6(2):122–136. 10.52326/jss.utm.2023.6(2).11

[22] Thinane JS. Religious perspectives on vaccination: Mandatory Covid-19 vaccine for SA churches. Pharos J Theol [serial online]. 2022 [cited 2024 Feb 10];103. Available from: https://www.pharosjot.com

[23] Oyo-Ita A, Bosch-Capblanch X, Ross A, et al. Effects of engaging communities in decision-making and action through traditional and religious leaders on vaccination coverage in Cross River State, Nigeria: A cluster-randomised control trial. PLoS One. 2021;16(4):e0248236. 10.1371/journal.pone.0248236 33861742 PMC8051768

[24] Ruijs CD, Kerkhof AJ, Van der Wale G, Onwuteaka-Philipsen BD. Symptoms, unbearability and the nature of suffering in terminal cancer patients dying at home: A prospective primary care study. BMC Fam Pract. 2013;14:201. 10.1186/1471-2296-14-201 24373224 PMC3877870

[25] Wirsiy FS, Nkfusai CN, Ako-Arrey DE, Dongmo EK, Manjong FT, Cumber SN. Acceptability of COVID-19 vaccine in Africa. Int J MCH AIDS. 2021;10(1):134–138. 10.21106/ijma.482 33868778 PMC8039868

[26] Ekwebelem OC, Yunusa I, Onyeaka H, Ekwebelem NC, Nnorom-Dike O. COVID-19 vaccine rollout: Will it affect the rates of vaccine hesitancy in Africa? Public Health. 2021;197:e18. 10.1016/j.puhe.2021.01.010 33714553 PMC7843135

[27] Afolabi AA, Ilesanmi OS. Dealing with vaccine hesitancy in Africa: The prospective COVID-19 vaccine context. Pan Afr Med J. 2021;38:3. 10.11604/pamj.2021.38.3.27401 33520072 PMC7825371

[28] Coates EA, Waisbord S, Awale J, Solomon R, Dey R. Successful polio eradication in Uttar Pradesh, India: The pivotal contribution of the Social Mobilization Network, an NGO/UNICEF collaboration. GlobHealth Sci Pract. 2013;1(1):68–83. 10.9745/GHSP-D-12-00018 PMC4168556 25276518

[29] Harapan H, Shields N, Kachoria AG, Shotwell A, Wagner AL. Religion and measles vaccination in Indonesia, 1991–2017. Am J Prevent Med. 2021;60(1 Suppl. 1):S44–S52. 10.1016/j.amepre.2020.07.029 PMC7894975 33189503

